# Generalizing a mathematical model of prion aggregation allows strain coexistence and co-stability by including a novel misfolded species

**DOI:** 10.1007/s00285-018-1280-4

**Published:** 2018-08-16

**Authors:** Paul Lemarre, Laurent Pujo-Menjouet, Suzanne S. Sindi

**Affiliations:** 1 School of Natural Sciences, University of California, Merced, 5200 North Lake Road, Merced, CA 95343 USA; 2 Institut Camille Jordan, Université de Lyon, Université Claude Bernard Lyon 1, CNRS UMR 5208, 43 blvd. du 11 novembre 1918, 69622 Villeurbanne cedex, France; 3 Team Dracula, INRIA, 69603 Villeurbanne cedex, France; 4 Applied Mathematics School of Natural Sciences, University of California, Merced, 5200 North Lake Road, Merced, CA 95343 USA

**Keywords:** Coexistence, Nucleated polymerization, Prions, Protein aggregation, Protein misfolding, Strains, Subunits, 34D20, 34D23, 37N35, 92C40, 92C60

## Abstract

Prions are proteins capable of adopting misfolded conformations and transmitting these conformations to other normally folded proteins. Prions are most commonly known for causing fatal neurodegenerative diseases in mammals but are also associated with several harmless phenotypes in yeast. A distinct feature of prion propagation is the existence of different phenotypical variants, called strains. It is widely accepted that these strains correspond to different conformational states of the protein, but the mechanisms driving their interactions remain poorly understood. This study uses mathematical modeling to provide insight into this problem. We show that the classical model of prion dynamics allows at most one conformational strain to stably propagate. In order to conform to biological observations of strain coexistence and co-stability, we develop an extension of the classical model by introducing a novel prion species consistent with biological studies. Qualitative analysis of this model reveals a new variety of behavior. Indeed, it allows for stable coexistence of different strains in a wide parameter range, and it also introduces intricate initial condition dependency. These new behaviors are consistent with experimental observations of prions in both mammals and yeast. As such, our model provides a valuable tool for investigating the underlying mechanisms of prion propagation and the link between prion strains and strain specific phenotypes. The consideration of a novel prion species brings a change in perspective on prion biology and we use our model to generate hypotheses about prion infectivity.

## Introduction

Prion diseases are a class of neurodegenerative disorders in mammals associated with a change in the folded shape (conformation) of the protein PrP. This misfolded conformation (PrP^Sc^) is believed to induce transformation of proteins in the normal conformation (PrP^C^), and proteins under this abnormal state may aggregate (Bolton et al. 1982; Collinge 2001; Aguzzi and Polymenidou 2004; Tuite and Serio 2010). Prions have also been discovered in yeast (Tuite and Cox 2003), where they are mostly associated with harmless epigenetic phenomena (Lindquist and Newby 2013). The dynamics of prion propagation have been studied extensively during the past decades, using experimental cases in mammals and yeast on one hand (Sindi and Serio 2009; Tuite and Cox 2003; Tuite and Serio 2010), and mathematical modeling on the other hand (Sindi 2017; Greer et al. 2006; Prüss et al. 2006; Masel et al. 1999). One feature of prion propagation that remains poorly understood theoretically is the ability of prions to develop a variety of infectious agents with different phenotypical properties, also called *strains* (Tanaka et al. 2006; Derdowski et al. 2010; Collinge and Clarke 2007; Colby and Prusiner 2011; Hunter et al. 1986). These differences are assumed to be associated with different conformations of the protein, which are then associated with strain-specific biochemical properties (Tanaka et al. 2006; Weissman et al. 2004; Lindquist and Krishnan 2005). Mounting evidence suggests that strains can coexist and compete, but it is unclear what drives their interactions (Shikiya et al. 2010; Bradley et al. 2002; Le Dur et al. 2017). We note that the phenomenon of strains applies not only to prion diseases, but also to other neurodegenerative disorders caused by *prion-like mechanisms* such as Alzheimer’s or Parkinson’s disease (Watts et al. 2014; Cohen et al. 2015).

Previous mathematical studies of spontaneous prion formation have led to widespread acceptance of the *nucleated polymerization model* (Lansbury and Caughey 1995), which has been formulated under the assumption of either discrete or continuous aggregate sizes (Masel et al. 1999; Greer et al. 2006; Doumic et al. 2009; Davis and Sindi 2015). This model indeed reproduces qualitative and quantitative aspects of prion infection (Prüss et al. 2006; Masel et al. 1999), all the while being analytically tractable (Engler et al. 2006). However, as we show here, this model does not explain the interactions between prion strains observed *in vivo* and *in vitro*. In particular, various studies demonstrate that strains may coexist in both yeast (Strbuncelj 2009) and mammals (Polymenidou et al. 2005) but this is not supported by the nucleated polymerization model. While many mathematical studies have generalized the classical model to consider strain specific phenomena, such as (Calvez et al. 2010) who modeled aggregate size-dependent rates, there has been remarkably little mathematical work considering interactions between prion strains. A recent generalization of the nucleated polymerization model showed strain coexistence was possible in yeast when fragmentation was mediated by the molecular chaperone Hsp104 (Davis and Sindi 2016b). However, because chaperones are not known to be part of the *in vivo* dynamics of mammalian prions, we wanted to identify chaperone independent mechanisms that could explain coexistence of prion strains in both yeast and mammalian systems.

In this work, we generalize the nucleated polymerization model by including a biologically motivated species we term subunits. More specifically, we add a conformational change step prior to polymerization, called *templating* and driven by subunits (Igel-Egalon et al. 2017, 2018). Different studies have suggested similar approaches before (Alvarez-Martinez et al. 2011; Hingant et al. 2014), but not in the specific case of strain interaction and coexistence. We note that our model exhibits qualitatively distinct behavior from the classical nucleated polymerization model. Most importantly, it enables the *coexistence* of different strains (multiple strains present stably together), but it also allows for simultaneous *co-stability* of different equilibria (dependency on initial conditions). These two features will be our main focus throughout the study. Note that our model does not depict the spontaneous formation of prion aggregates because this process happens on a distinct time-scale. The appearance of the first stable prion nucleus is typically modeled as the first-arrival time of a stochastic process, as in D’Orsogna et al. (2012), Yvinec et al. (2012, 2016), Davis and Sindi (2016a). In this work, our analytical and numerical results concern the propagation of prions from an initially established pool of aggregates.

In Sect. 2 we review the nucleated polymerization model and discuss its limitations with respect to multiple strain behavior. Section 3 introduces our model, with primary analytical results on its behavior in Sects. 3.1 and  3.2, a bifurcation analysis in Sect. 3.3 and a detailed analytical study of the two-strain case in Sect. 3.4. We end this section with numerical validations in Sect. 3.5. Finally, we discuss several implications of our model in the context of open questions in prion biology in Sect. 4.1, we identify the limitations and upcoming challenges in Sect. 4.2 and we present some justification for our choice over other possible modeling ideas in Sect. 4.3.

## Background: the nucleated polymerization model can only predict single strain dominance

### Nucleated polymerization model

**Fig. 1:**
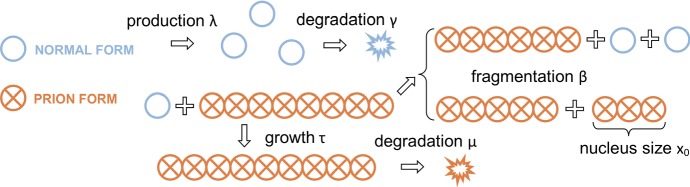
Nucleated polymerization model with a single prion strain, illustrated with a nucleus size of $x_0=3$. The normal form monomers (empty circles) appear with a production rate $\lambda$ and are degraded with a rate $\gamma$. They are recruited by prion aggregates (crossed circles) with rate $\tau$. Each link in an aggregate may break with probability $\beta$, and an aggregate below the nucleus size $x_0$ is disassembled into normal monomers. The aggregates are degraded with rate $\mu$

We will first present the nucleated polymerization model describing prion dynamics when only a single strain is present. In this model, all protein monomers are either in the normally folded state or in the misfolded (prion) state. In the classical nucleation theory, the misfolded form of the protein is only stable when it belongs to an aggregate of size larger than a critical nucleus $x_0$ (Masel et al. 1999). The dynamics of the aggregates and normally folded proteins evolve through several biochemical processes as depicted in Fig. 1. Aggregates may increase in size when proteins in the normally folded state are converted to the misfolded form and incorporated into aggregates, this is called polymerization (Bolton et al. 1982; Collinge 2001). Aggregates may increase in number by fragmenting. In the original formulation, a prion aggregate is assumed to be a linear array of misfolded protein monomers. As such, polymerization can only occur as the ends of an aggregate interact with normal monomers at rate $\tau$. Similarly, fragmentation is assumed to occur at rate $\beta$ between any two adjacent misfolded monomers in an aggregate. In accordance with the nucleation assumption, if a fragmentation event creates an aggregate with size smaller than the nucleus size $x_0$, it is immediately disassembled and the constituent proteins return to the normally folded state. Finally, normal monomers are produced with a source rate $\lambda$ and degraded with rate $\gamma$ and aggregates are degraded with rate $\mu$.

In the mathematical literature, both continuous and discrete size aggregate distributions have been considered (Masel et al. 1999; Davis and Sindi 2015; Greer et al. 2006; Prüss et al. 2006). While the results and approaches taken are similar, in what follows we use the continuous (in aggregate size) formulation studied in Greer et al. (2006) and Prüss et al. (2006) because our interest is mainly in qualitative study of the dynamics. In this case, the state space dynamics are governed by ordinary differential equation for *V*, the concentration of normally folded monomers and a partial differential equation, a transport equation, governing the aggregate size distribution *u*(*x*, *t*) as shown below: 
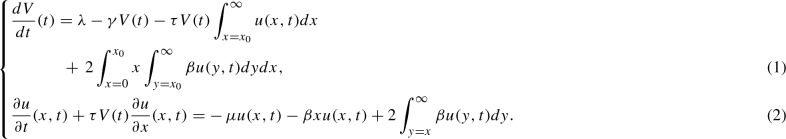
 This system is to be completed with boundary conditions $u(x_0,t)=0$ and $\lim _{x \rightarrow \infty }u(x,t)=0$ for $t\ge 0$, as well as initial conditions $V(t=0)=V_0$ and $u(x,0)=u_0(x)$ for $x\in [x_0,\infty [$. In many cases, we are interested in the qualitative behavior of the system, such as whether misfolded protein will exist asymptotically, rather than the time-evolving aggregate density. To facilitate this analysis, we convert the Eqs. (1)–(2) to a system of ordinary differential equations using moment closure. More specifically, for $t\ge 0$ we define $U(t) =\int _{x=x_0}^\infty u(x,t)dx$ to be the number of aggregates, the zeroth moment of the aggregate density, and $P(t)=\int _{x=x_0}^\infty xu(x,t)dx$ to be the total mass of aggregated protein, the first moment of the aggregate density. We obtain the following system of ordinary differential equations involving the moments *U*(*t*), *P*(*t*) and the population of normal monomers *V*(*t*): 
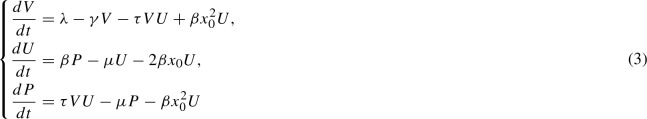


where $t \ge 0$ and initial conditions $V(0) \ge 0, U(0) \ge 0$ and $P(0) \ge x_0 U(0)$.

As has been discussed in prior studies (Prüss et al. 2006; Greer et al. 2006) this system asymptotically approaches one of two possible steady-states. Either the *Disease Free Equilibrium (DFE)* corresponding to the presence of only the normally folded protein, $(V,U,P) = (\lambda /\gamma ,0,0)$ or the *Endemic Steady-State (ESS)* where all three quantities are non-negative indicating the asymptotic stability of prion aggregates:

4 $$\begin{aligned} (V,U,P)=\left( \frac{(\mu +\beta x_0)^2}{\beta \tau },\frac{\lambda \beta \tau -\gamma (\mu +\beta x_0)^2}{\mu \tau (\mu +2\beta x_0)},\frac{\lambda \beta \tau -\gamma (\mu +\beta x_0)^2}{\beta \mu \tau } \right) . \end{aligned}$$

The stability of the ESS depends on the basic reproductive number of the prion strain,

5 $$\begin{aligned} R_0 = \frac{\lambda \beta \tau }{\gamma \left( \mu +\beta x_0\right) ^2}. \end{aligned}$$

When $R_0 > 1$, as long as some aggregates are initially present (i.e., $U(0)>0$), the system will approach the ESS. These global stability results are proved in Greer et al. (2006). This basic reproductive number plays the role commonly observed in epidemiological models (Brauer and Castillo-Chavez 2010).

### Multi-strain nucleated polymerization model

As mentioned in the introduction, we are interested in the behavior of multiple prion strains and so we next consider a natural generalization of this model to multiple strains. Similarly to Tanaka et al. (2006), we assume the same biochemical processes apply to all strains and that aggregates corresponding to distinct prion strains do not directly interact. Under these assumptions, strains each have their own aggregates and the aggregates dynamics are governed by strain-specific biochemical rates. That is, in a system with *N* different strains ($N>1$) we have strain-specific parameters $x_i,\tau _i,\beta _i$ and $\mu _i$ for $i \in \{1, \ldots N\}$. The only difference from the nucleated polymerization model is that strains compete for the same resource, the normally folded monomers. As such, we obtain a similar moment closure for each strain. The system consists of a single equation for the population of normal monomers, *V*(*t*), and one for each moment of each strain, $U_i(t), P_i(t)$
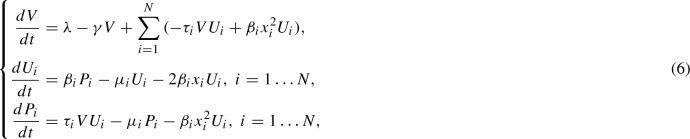


where $N \ge 1$, $t\ge 0$ and initial conditions where for each strain $U_i(0) \ge 0, P_i(0) \ge x_i U_i(0).$

Let us now consider the different steady-states possible in our multi-strain nucleated polymerization model given by System (6). Note that different steady-states are possible depending on the parameters. First, the *Disease-Free Equilibrium*, when all prion strains become extinct, always exists and is given by

$$\begin{aligned}(V,U_1,P_1,\ldots ,U_N,P_N)=(\lambda /\gamma ,0,0,\ldots ,0,0).\end{aligned}$$

Then for each strain *i*, $i \in \{1,\ldots N\}$, there is a *Strain-Specific Endemic Steady-State* corresponding to the asymptotic presence of strain *i* only. It is given by

$$\begin{aligned}&(V,U_1,P_1,\ldots ,U_i,P_i,\ldots ,U_N,P_N)\\&\quad =\Big (\frac{(\mu _i+\beta _i x_i)^2}{\beta _i\tau _i},0,\ldots ,0,\frac{\lambda \beta _i\tau _i -\gamma (\mu _i+\beta _i x_i)^2}{\mu _i \tau _i(\mu _i+2\beta _i x_i)},\frac{\lambda \beta _i\tau _i-\gamma (\mu _i+\beta _i x_i)^2}{\mu _i\beta _i\tau _i},0,\ldots ,0\Big ). \end{aligned}$$

We note that the nonzero quantities in the steady-state correspond exactly to the nucleated polymerization endemic steady-state for the strain in isolation (Eq. (4)). The strain-specific endemic steady-state is biologically feasible (i.e., all concentrations are positive) only when $\frac{\lambda }{\gamma }\beta _i \tau _i>(\mu _i+\beta _i x_i)^2$. As we did for the single strain case, and has been done in prior studies (Prüss et al. 2006; Greer et al. 2006), we define a strain-specific basic reproductive number

7 $$\begin{aligned} R_0^i=\frac{ \lambda \beta _i \tau _i}{ \gamma (\mu _i+\beta _i x_i)^2}, \end{aligned}$$

and note that biological feasibility of the strain-specific endemic steady-state corresponds to exactly $R_0^i>1$.

Next, we consider steady-states where multiple strains exist together. We define such steady-states to be *Coexistence Steady-States*. We note that for each strain at steady-state, the following strain-specific relation on the normally folded protein monomer density must be satisfied:

8 $$\begin{aligned} V=\frac{(\mu _i+\beta _i x_i)^2}{\beta _i \tau _i} = \frac{\lambda }{\gamma } \frac{1}{R_0^i}. \end{aligned}$$

As such, only strains with the same basic reproductive number can exist together at steady state. This means that as long as strains have different steady-state normal monomer concentrations, they cannot coexist at steady-state in our multi-strain model. Because the reproductive number of a strain $R_0^i$ is a function of the strain-specific biological parameters, it is highly unlikely that these $R_0^i$ coincide exactly. Moreover, even if by chance two strains had an identical $R_0^i$, coexistence would not be robust as the slightest perturbation of one parameter would completely remove the possibility of a coexistence equilibrium.

In addition, much like the original nucleated polymerization model, only one steady-state at a time will be asymptotically stable. The behavior of this multi-strain model (6) is driven by the following theorem.

#### Theorem 1

Assume all parameters $\lambda ,\gamma$ and $\tau _i,\beta _i,x_i,\mu _i$ for $i=1\ldots N$ are positive. Then the system (6) admits a unique positive solution for each initial condition taken in

$$\begin{aligned} X=\{ (V,U_1,P_1,\ldots ,U_N,P_N) \in {\mathbb {R}}^{2N+1}:V,U_1,P_1-x_1U_1,\ldots ,U_N,P_N-x_N U_N \ge 0\}. \end{aligned}$$

Further suppose that the quantities $R_0^i=\frac{\lambda \beta _i\tau _i}{\gamma (\mu _i+\beta _i x_i)^2}$ are distinct and define

$$\begin{aligned}{\mathscr {R}}_0= \underset{i=1\ldots N}{\max }\left\{ R_0^i \right\} .\end{aligned}$$

If ${\mathscr {R}}_0\le 1$, the disease-free equilibrium $(\lambda /\gamma ,0,0,\ldots ,0,0)$ is globally asymptotically stable on *X*.

If ${\mathscr {R}}_0>1$, suppose Strain 1 verifies this maximum (renumbering the strains if necessary), there exists an endemic equilibrium for each strain with $R_0^i>1$ (including Strain 1). The equilibrium involving only Strain 1 is given by

$$\begin{aligned} \left( \frac{(\mu _1+\beta _1 x_1)^2}{\beta _1\tau _1},\frac{\lambda \beta _1\tau _1 -\gamma (\mu _1+\beta _1 x_1)^2}{\mu _1 \tau _1(\mu _1+2\beta _1 x_1)},\frac{\lambda \beta _1\tau _1-\gamma (\mu _1+\beta _1 x_1)^2}{\mu _1\beta _1\tau _1},0,0,\ldots ,0,0\right) , \end{aligned}$$

and is globally asymptotically stable on $\left\{ (V,U_1,P_1,\ldots ,U_N,P_N)\in X: U_1>0 \text { and } P_1>0 \right\}$. Note that if $U_1(0)=P_1(0)=0$, the outcome will be given by the same theorem without considering Strain 1.

#### Proof

The existence and uniqueness of solutions is proved in the same fashion as in the single strain case, with direct adaptation of the proof provided in Prüss et al. (2006). The global results rely on Lyapunov functions, and the proof is presented in Appendix A. $\square$

### Biological interpretation and limitations

In the previous section, we demonstrated that the natural generalization of the nucleated polymerization model to multiple strains allows for at most one strain to persist, and the steady-state associated with this strain will be globally asymptotically stable. However, this is not consistent with biological studies suggesting a diversity of behaviors is possible with prion strains. Before we develop our template-assistance model, we first describe biological behavior observed both in yeast and mammalian prion systems that the previously introduced model does not support.

#### Coexistence of different strains cannot be explained

Numerous experiments demonstrate that distinct prion strains may be present stably at the same time in a host, even though they are associated with dramatically different disease properties (Polymenidou et al. 2005; Le Dur et al. 2017; Langenfeld et al. 2016). As emphasized in Sect. 2.2, coexistence equilibria for the multi-strain nucleated polymerization model only exist when strains have exactly the same values of $R_0^i$. We note that this is biologically unlikely because it describes a set of measure zero in parameter space. One could argue that, even if the coexistence steady-state is impossible, multiple strains could be present together for a long time before one takes over. This indeed happens when the $R_0^i$ values are very close to each other. However, judging from phenotype differences between strains observed *in vivo* in mammals as well as yeast (Le Dur et al. 2017; Strbuncelj 2009), it seems likely that these coexisting strains possess very different propagation properties. Under these conditions, the multi-strain nucleated polymerization model predicts the fast takeover by one strain. This is the first main flaw in the classical model, and its generalization with multiple strains.

#### Co-stability of different steady-states cannot be explained

In some experimental studies for mammalian prions, the observable outcome appears to vary based only on changes in the inoculum, *i.e.* the initial condition. For example see Le Dur et al. (2017), where diluting the inoculum changes the strain which dominates, and repeating the same experiment (with possibly slight variations in the inoculum) yields different possible outcomes. In Langenfeld et al. (2016), superinfection leads to different outcomes depending on the delay between the first inoculation (with one strain) and the second (with another strain). Theorem 1 spotlights a weakness of the model regarding these observations, namely that its behavior is global. Regardless of the case, there is only one equilibrium at a time that is globally asymptotically stable. This means that with this model, the outcome does not depend at all on the initial conditions. In particular, in the endemic case, one strain always takes over the other ones.

Although the nucleated polymerization model is clearly insufficient to investigate multiple strain phenomena, it is still coherent with experimental data in the single strain case, and it is analytically well understood (Prüss et al. 2006; Engler et al. 2006; Greer et al. 2006). Based on these considerations, we will use the nucleated polymerization model as a “building block” towards a model to study multiple strain phenomena, as we explain in Sect. 3.

## The template assistance model

**Fig. 2:**
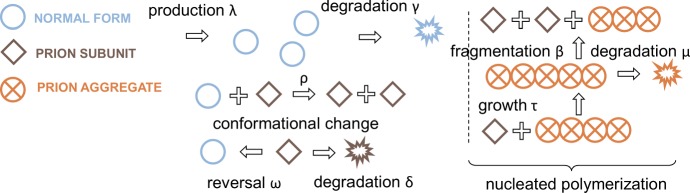
Template assistance mechanism illustrated for a single prion strain. The normal monomers (empty circles) are converted by the subunits (squares) by direct interaction at rate $\rho$. This first conformational change is reversed spontaneously at rate $\omega$, and the subunits are also degraded at rate $\delta$. The aggregates are formed of subunits, and the dynamics driving the interaction between aggregates and subunits are those of the nucleated polymerization model (similarly to Fig. 1, with subunits playing the role of monomers). The aggregates are degraded at rate $\mu$

Through mathematical investigation, we identified different generalizations of the nucleated polymerization model that could allow for multiple strain coexistence and co-stability (see Sect. 1 for precise definition). The simplest idea that appeared both encouraging and biologically consistent (see Sect. 4.3 for a parallel with ecological populations and a discussion that supports our modeling choice) was to add a step prior to polymerization, and to consider an additional biochemical species. This species represents subunits (potentially small oligomers) that are responsible for the templating activity (monomer conversion). The aggregation of subunits follows the nucleated polymerization dynamics and Fig. 2 illustrates the principles of such a model.

We note that our concept of subunits has a biological basis. Indeed recent studies suggest that for mammalian prions, aggregates are in a kinetic equilibrium with subunits (Igel-Egalon et al. 2017, 2018). It is still not clear how these subunits are involved in templating *in vivo*, but we here include them as the main templating agent. In yeast, there is also evidence of *in vitro* prion formation following different pathways (Sharma et al. 2017). This modeling approach has been considered in previous studies (Hingant et al. 2014; Yvinec 2012), although never with a continuous and deterministic formulation. Additionally, this modeling approach has never been used to investigate interactions between multiple prion strains. We will now formally introduce this generalization and investigate the behavior of our model with respect to multiple strain coexistence and interaction.

### Model formulation for a single strain and primary results

We first introduce our template assistance model for a single strain. In our formulation we preserve the notation introduced for the nucleated polymerization model in Sect. 2, but we now introduce a new species, namely the subunit population *S*(*t*) at time $t\ge 0$. As shown in Fig. 2, the initial conformational change is reversible, and subunits return to the normal form at rate $\omega$. They are degraded with a rate $\delta$, most likely $\delta <\gamma$ representing the fact that the subunits are more resistant than normal monomers. As illustrated in Fig. 2, the subunits are formed by direct interaction between subunits and monomers with speed $\rho V f(S)$. The function *f* models the efficiency of subunits to convert monomers. Our choice is

$$\begin{aligned} f(S)=S\frac{S}{K+S}, \end{aligned}$$

which depicts a Michaelis–Menten process for the conformational change reaction (a mathematical justification for this choice will come later). Essentially, this reaction is not instantaneous, and the population of subunits *S* has to be above a certain threshold before reaching full efficiency (*K* representing the density at which half the maximal efficiency is reached). One could also say that the fraction of actively involved subunits is given by a Hill function (here of order 1 for simplicity), which is a commonly used class of functions to model enzyme-mediated biochemical kinetics (see for instance Segel and Edelstein-Keshet 2013, Chapter 8). A linear templating rate would prevent co-stability as shown in Sect. 3.2, and the non-linearity introduced appears a sufficient condition for co-stability although we can only show it numerically in Sects. 3.3 and 3.5. Proving it analytically remains an open problem that we will investigate in future work. In addition, there is precedence for considering Michaelis–Menten dynamics in prion kinetics, Milto et al. (2013) considered fibril elongation occurring as an enzyme-mediated reaction. This is not our choice here, but we suggest the templating by subunits is a cooperative reaction. The elongation rate could indeed be modified to obtain the same behavior as we obtain here, but it would require inconsistent hypotheses with the biological context we focus on, see Sect. 4.3 for a detailed discussion. As long as a quantitative study is not experimentally possible, to the best of our knowledge (see Sect. 4.2), we consider the simplest option for the elongation rate.

The full aggregate dynamics are now defined by two ordinary differential equations (for *V*(*t*) and *S*(*t*)) and a single partial differential equation for *u*(*x*, *t*), which will be identical to Eq. (2) but with *S*(*t*) replacing *V*(*t*) in the transport term. Under these assumptions, we obtain a similar moment closure to the equations of the nucleated polymerization model 
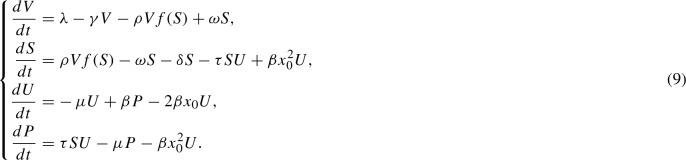


Similarly to the nucleated polymerization model, the System (9) has a unique positive solution. In particular, we prove the following Lemma.

#### Lemma 1

When the parameters $\lambda ,\gamma ,\beta ,\tau ,\mu ,x_0,\rho ,\omega ,\delta , K$ are all positive, the system (9) admits an unique positive solution for each initial condition taken in

$$\begin{aligned} X=\{(V,S,U,P)\in {\mathbb {R}}^4: V,S,U,P-x_0 U\ge 0\}. \end{aligned}$$

#### Proof

It is simple to verify that *X* is positively invariant. Furthermore, for $t>0$, $V(t)+S(t)+P(t)$ is bounded between 0 and $\frac{\lambda }{\epsilon }+(V(0)+S(0)+P(0))e^{-\epsilon t}$, with $\epsilon =\min \{\gamma ,\delta ,\mu \}$. The proof is concluded as in Prüss et al. (2006). $\square$

### Steady-state analysis

We will next investigate the existence and, when possible, the linear stability of steady-states of our system. Because it will be useful in our analysis, we state here the Jacobian matrix of System (9) for clarity

$$\begin{aligned}&{\mathbf {J}}(V,S,U,P)\\&\quad =\left( \begin{matrix} -\gamma -\rho f(S) &{}\quad \omega - \rho Vf'(S) &{}\quad 0 &{}\quad 0 \\ \rho f(S) &{}\quad \rho Vf'(S)-\omega -\delta -\tau U &{} \quad -\tau S +\beta x_0^2 &{}\quad 0 \\ 0 &{}\quad 0 &{}\quad -\mu -2\beta x_0 &{}\quad \beta \\ 0 &{}\quad \tau U &{}\quad \tau S-\beta x_0^2 &{}\quad -\mu \\ \end{matrix}\right) . \end{aligned}$$

#### Proposition 1

The disease-free equilibrium exists for any choice of positive parameters, and is given by

$$\begin{aligned} (V,S,U,P)=\left( \frac{\lambda }{\gamma },0,0,0\right) . \end{aligned}$$

It is locally stable if and only if

$$\begin{aligned} \frac{\lambda }{\gamma }f'(0) <\frac{\omega +\delta }{\rho }. \end{aligned}$$

In particular when $f'(0)=0$, as with the choice $f(S)=S^2/(K+S)$ introduced in Sect. 3.1, the disease-free equilibrium is locally stable for any choice of positive parameters.

#### Proof

A straightforward calculation yields the eigenvalues of the Jacobian matrix at this points $\{ -\gamma , -\mu -\beta x_0, -\mu -\beta x_0, \frac{\lambda }{\gamma }f'(0)-\omega -\delta \}$. For positive parameters, the local stability depends only on the last one, and it is negative when the condition expressed above is verified. $\square$

The local stability of the disease-free equilibrium is the new feature that will enable co-stability of different equilibria. Any other function verifying $f'(0)=0$ would also yield the same property, but our choice was to introduce as few parameters as possible. On the contrary, we can show that a linear templating rate would not provide co-stability, as explained at the end of this section. A second type of steady-state is possible, when subunits are present but no aggregates.

#### Proposition 2

There exists at most two subunits-only equilibria, and they are given by

$$\begin{aligned} (V,S,U,P)=\left( \frac{\lambda }{\gamma }-\frac{\delta }{\gamma }S_{+/-},S_{+/-},0,0\right) \end{aligned}$$

with

$$\begin{aligned} S_{+/-}=\frac{1}{2}\left( \frac{\lambda }{\delta }-\frac{\omega +\delta }{\rho }\frac{\gamma }{\delta }\right) \pm \sqrt{\left( \frac{1}{2}\left( \frac{\lambda }{\delta }-\frac{\omega +\delta }{\rho }\frac{\gamma }{\delta }\right) \right) ^2-\frac{\omega +\delta }{\rho }\frac{\gamma }{\delta }K}. \end{aligned}$$

These equilibria are feasible (positive values) when the following condition is met

$$\begin{aligned} \frac{\lambda }{\gamma }>\frac{\omega +\delta }{\rho } +2\frac{\delta }{\gamma }\sqrt{\frac{\omega +\delta }{\rho }\frac{\gamma }{\delta }K}. \end{aligned}$$

The equilibrium associated with $S_{-}$ is always locally unstable. The equilibrium associated with the higher value is locally stable if and only if

$$\begin{aligned} S_+ < \frac{(\mu +\beta x_0)^2}{\beta \tau }. \end{aligned}$$

#### Proof

If we impose $U=0$ and $P=0$, the remaining two equations on *V* and *S* lead to the equation $S^2+\left( \frac{\omega +\delta }{\rho }\frac{\gamma }{\delta }-\frac{\lambda }{\delta }\right) S+\frac{\omega +\delta }{\rho }\frac{\gamma }{\delta }K=0.$ This gives directly the expression of the two possible values for *S*, and the condition for them to be real and positive. Their stability is once again analyzed through the Jacobian matrix. For both of the points $S_{+/-}$, a pair of eigenvalues is given by $\left( -\mu -\beta x_0+\sqrt{\tau \beta S_{+/-}},-\mu -\beta x_0 -\sqrt{\tau \beta S_{+/-}}\right)$. These eigenvalues are both negative when $S_{+/-}<\frac{(\mu +\beta x_0)^2}{\beta \tau }$. The other pair of eigenvalues are the roots of a second degree polynomial (not shown for simplicity), and after simplification we show that they are negative when $S_{+/-}>\sqrt{\frac{\omega +\delta }{\rho }\frac{\gamma }{\delta }K}$. Recalling the values of $S_+$ and $S_-$, we see that this second condition is never met by $S_-$ and always met by $S_+$. Hence the condensed result in the proposition. $\square$

This result shows that the two subunits-only equilibria emerge through a saddle-node bifurcation, and that only the one associated with a higher *S* value might be stable, with condition related to the last equilibrium. See Sect. 3.3 for a numerical illustration of this bifurcation.

#### Proposition 3

The endemic steady-state $(V^*,S^*,U^*,P^*)$ is defined by the following relations

$$\begin{aligned} S^*&=\frac{(\mu +\beta x_0)^2}{\beta \tau }, \\ V^*&=\frac{\lambda +\omega S^*}{\gamma + \rho f(S^*)}, \\ U^*&=\frac{1}{\tau S^*-\beta x_0^2}(\rho V^* f(S^*) -\omega S^* -\delta S^*),\\ P^*&=\frac{\mu +2\beta x_0}{\beta } U^*. \end{aligned}$$

This equilibrium is feasible (positive values) when

$$\begin{aligned} \lambda > \delta S^* + \frac{\omega +\delta }{\rho }\frac{\gamma S^*}{f(S^*)}. \end{aligned}$$

#### Proof

The feasibility mainly relies on $U^*>0$, which with the value of $V^*$ reduces to the condition expressed above (noticing that $\tau S^*-\beta x_0^2=\mu (\mu +2\beta x_0)/\beta > 0$). $\square$

Recalling the conditions for the feasibility of the two subunits-only equilibria, we note that the endemic steady-state exists when the associated subunit density $S^*$ is comprised between the two subunits-only values $S_{+/-}$. Indeed the condition expressed in Proposition 3 implies (but is not equivalent to) $f(S^*)>\frac{\omega +\delta }{\rho }\frac{\gamma S^*}{\lambda -\delta S^*}$, and the two subunit-only equilibrium are the solutions that verify equality in this relation. We investigate the stability of this equilibrium numerically through a bifurcation analysis in the next section.

#### Remark 1

Although we cannot show analytically that the condition $f'(0)=0$ yields co-stability of different equilibria, we can show that when *f* is linear there is no co-stability possible. Indeed, if $f(S)=S$, which is equivalent to $K=0$ in all the developments above, there is only one subunits-only equilibrium associated with the subunit density $\hat{S}=\frac{\lambda }{\delta }-\frac{\omega +\delta }{\rho }\frac{\gamma }{\delta }$. The stability condition for the disease-free equilibrium is reduced to $\frac{\lambda }{\gamma }<\frac{\omega +\delta }{\rho }$ or equivalently $\hat{S}<0$, which shows that these two equilibria cannot be both feasible and stable at the same time. The subunits-only equilibrium is in turn stable as long as $\hat{S}<\frac{(\mu +\beta x_0)^2}{\beta \tau }=S^*$ (with the notation of Proposition 3. However, the feasibility condition of the endemic equilibrium is now reduced to $V^*>\frac{\omega +\delta }{\rho }$ ($V^*$ defined in Proposition 3) or equivalently $\frac{\lambda }{\delta }-\frac{\omega +\delta }{\rho }\frac{\gamma }{\delta }=\hat{S}>S^*$ after simplification. This proves that the subunits-only equilibrium and the endemic equilibrium cannot be both feasible and stable at the same time. Essentially, when *f* is linear there are only three possible equilibria and they appear through a series of transcritical bifurcations, thus there can be no stability.

### Numerical bifurcation analysis

**Fig. 3:**
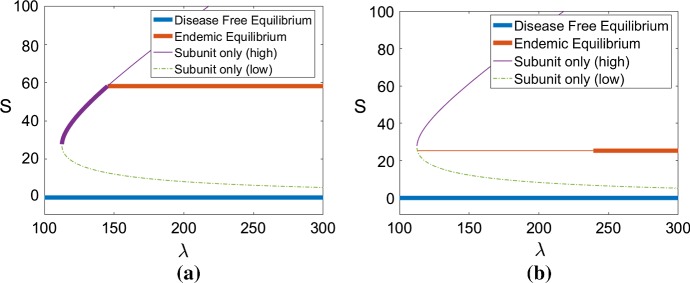
Bifurcation diagram obtained for the steady-state subunit populations *S* when the monomer source, $\lambda$, varies for two values the polymerization rate $\tau$. The other parameters are set as in Table 1 (Strain 1). The four different equilibria are depicted (disease-free equilibrium, endemic equilibrium, higher subunit only equilibrium $(S_+)$, lower subunit only equilibrium $(S_-)$). A thin line indicates an unstable equilibrium, whereas a bold line indicates a locally stable equilibrium. **a**$\tau = 0.1$, **b**$\tau = 0.23$

As shown in Sect. 3.2, the subunits-only steady-states appear through a saddle node bifurcation (Proposition 2). The endemic steady-state appears when the associated subunit population $S^*$ crosses one of the two branches $S_+$ or $S_-$ (Proposition 3). If it appears through the higher branch, the high subunit only steady-state $S_+$ is stable at first, and then becomes unstable. If the endemic steady-state instead appears through the lower branch, none of the subunit only steady-states will be stable (see conditions in Sect. 3.2). These two scenarios are illustrated in Fig. 3. As the monomer source rate $\lambda$ increases, we can see the saddle-node bifurcation followed by the emergence of the endemic steady-state. This is a numerical illustration of the results proved in the previous section.

**Table 1:** Parameter definitions and values used for numerical simulations (unless specified otherwise)

Parameter	Definition	Value	
$\lambda$	Monomer source rate	1500	
$\gamma$	Monomer degradation rate	5	

		Strain 1	Strain 2
$\rho$	Conversion rate of normal monomers to subunits	5	1
$\omega$	Reconversion rate of subunits	1	1
*K*	Half-maximum concentration for the conversion kinetics	500	100
$\delta$	Degradation of subunits	2	2
$\tau$	Polymerization rate of subunits	0.1	0.2
$\beta$	Fragmentation rate of polymers	0.0003	0.1
$\mu$	Degradation rate of polymers	0.04	0.04
$x_0$	Nucleus size	6	6

The values are chosen arbitrarily, with magnitudes consistent with values from Masel et al. (1999), where time is expressed in *days*. The aggregate-associated quantities are in fibril number per volume unit

**Fig. 4:**
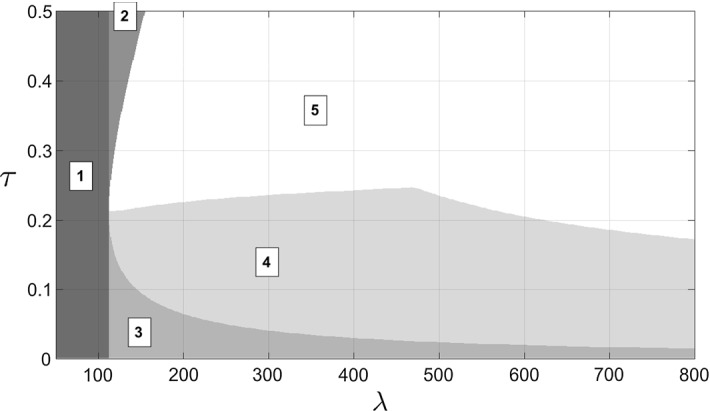
Two-parameter bifurcation diagram obtained for the monomer source rate $\lambda$ and the polymerization rate $\tau$, when the other parameters are described in Table 1 (Strain 1). The vertical line delimits the apparition of the subunit only equilibria, left of that line (*Region 1*) only the disease-free equilibrium exists and it is then globally stable (not proved). The branches delimit the existence of the endemic equilibrium. In *Region 2*, the subunit only equilibria exist but neither is stable. In *Region 3*, the subunit only equilibria exist and the higher one $(S_+)$ is locally stable. In-between the branches, the endemic steady-state exists, but it is only locally stable in *Region 4*. In *Region 5*, the endemic steady-state is unstable. The boundary between *Regions 4* and *5* corresponds to a Hopf bifurcation

When the endemic steady-state exists, it can be locally stable or unstable. Numerical exploration shows that it undergoes a Hopf bifurcation. This is illustrated with a numerical two-parameter bifurcation analysis in Fig. 4. We show dependence on two key parameters: the monomer synthesis rate $\lambda$ and the polymerization rate $\tau$. The bifurcation analysis is based on the properties of the Jacobian matrix at the endemic steady-state, and we characterize the presence of a Hopf bifurcation as when one eigenvalue crosses the imaginary axis with a non-zero imaginary part. Figure 4 illustrates regions of qualitatively distinct dynamics and the boundaries between these regions correspond to different bifurcation events. The endemic steady-state is only stable in Region 4, and the Hopf bifurcation occurs at the transition between Regions 4 and 5. In Region 5, only the disease-free equilibrium is locally stable. The solutions can either be attracted by this equlibrium or undergo stable oscillations. The exact nature of these oscillations is not known analytically, but the most important qualitative result here is that by crossing this Hopf bifurcation, and aggregate population can possibly be destabilized. The fact that oscillations have been observed experimentally (H. Rézaei, unpublished data) is another reason for interest in that behavior. Overall, the complexities of bifurcations in our model demonstrate the endemic steady-state is not always stable, and this is novel behavior to the nucleated polymerization model.

### Two-strain case

**Fig. 5:**
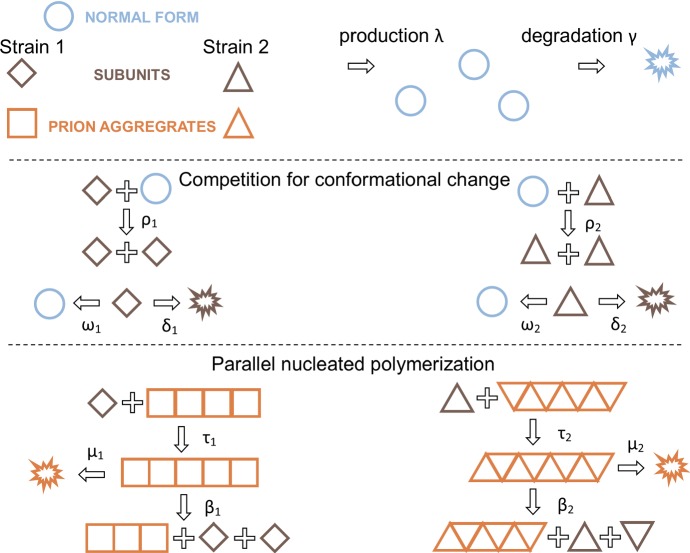
Template assistance mechanism illustrated with two different strains (squares and triangles). The strains compete for normal monomers through the interaction with subunits (with rates $\rho _1$ and $\rho _2$). The dynamics of each strain taken individually are then described as in Fig. 2

As we have emphasized throughout, our primary motivation in developing the template assistance model was to explore the system when multiple prion strains are present. We next generalize our model to two strains, as illustrated in Fig. 5. The corresponding generalization to System (9) is the following: 
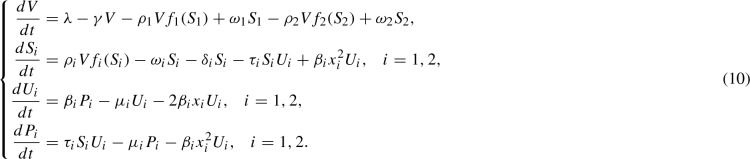
 The interaction functions, $f_i$, are the same as in System (9), but now with a strain specific value of $K_i$: $f_i(S)=S^2/(K_i+S)$.

As we will next detail, the behavior of this System (10) is dramatically different from that of the multi-strain nucleated polymerization model [System (6) presented in Sect. 2]. Our new system allows for coexistence of prion strains; that is, each strain may exist in the same conditions as single strains did Sect. 3.2. Furthermore, two strains may coexist under different configurations: both as subunits only, or one endemic and the other as subunits only (we will refer to this case as semi-endemic) or both endemic. We note that subunit only coexistence is impossible for general values of the parameters, with the same argument that was used to rule out coexistence equilibria in the multi-strain nucleated polymerization model in Sect. 2.2. Finally, numerical results suggest that semi-endemic coexistence is very unlikely to occur.

The coexistence steady-state is given by

$$\begin{aligned} S_i^*&=\frac{(\mu _i+\beta _i x_i)^2}{\beta _i\tau _i} \quad \text { for i =1,2}, \\ V^*&=\frac{\lambda +\omega _1 S_1^*+\omega _2 S_2^*}{\gamma +\rho _1 f_1(S_1^*)+\rho _2 f_2(S_2^*)},\\ U_i^*&=\frac{1}{\tau _i S_i^*-\beta _i x_i^2}(\rho _i V^*f_i(S_i^*)-\omega _i S_i^* -\delta _i S_i^*) \quad \text { for }i =1,2,\\ P_i^*&=\frac{\mu _i+2\beta _i x_i}{\beta _i}U_i^* \quad \text { for i =1,2}. \end{aligned}$$

Notice $\tau _i S_i^*=\mu _i(\mu _i+2\beta _i x_i)/\beta _i > 0$. Similarly to the single-strain case, this equilibrium exists when all values of the variables are positive yielding the conditions

$$\begin{aligned} f_i(S_i^*)>\frac{\omega _i +\delta _i}{\rho _i }\frac{S_i^*}{V^*} \text { for i}=1,2. \end{aligned}$$

While we were unable to simplify these conditions, they are easily verified for a given combination of parameters. In particular, we note that for any choice of kinetic rates for prion strains $(\beta _i, \tau _i, \omega _i, \rho _i)$, the coexistence steady-state can always be made to exist by increasing the monomer source rate $\lambda$ enough to satisfy the above conditions (because increasing $\lambda$ increases $V^*$ without changing the values of $S_1^*$ and $S_2^*$).

The second improvement of our System (10) over the multi-strain nucleated polymerization is that it allows for co-stability. As is the case with a single strain, the disease-free equilibrium is always locally stable (easily proved with the Jacobian matrix). Again as in the single-strain case, the co-stability of different types of equilibria (single-strain steady-states and coexistence steady-states) could not be proved analytically but can be observed through numerical exploration. As such, the outcome depends on the initial conditions as we will further investigate in the next section.

### Numerical results

In this section we provide a detailed numerical study of the behavior of the template assistance model we developed in the previous section. We first take care to verify that the behavior in the single strain case remains similar to the original nucleated polymerization model. We next study the dependency on initial condition when multiple steady-states are locally stable by numerically investigating the basins of attraction. Notice that in the different time evolution figures we produce, the evolution of *P*, the first moment of the aggregates distribution *i.e.* the total mass or number of aggregated monomers, is not displayed because it is very similar to the evolution of *U*, the zero-th moment of the distribution *i.e.* the total number of aggregates. However, *P* is on a different scale of values than the other variables so for the sake clarity we do not show it.

#### The dynamics of the nucleated polymerization model are conserved

**Fig. 6:**
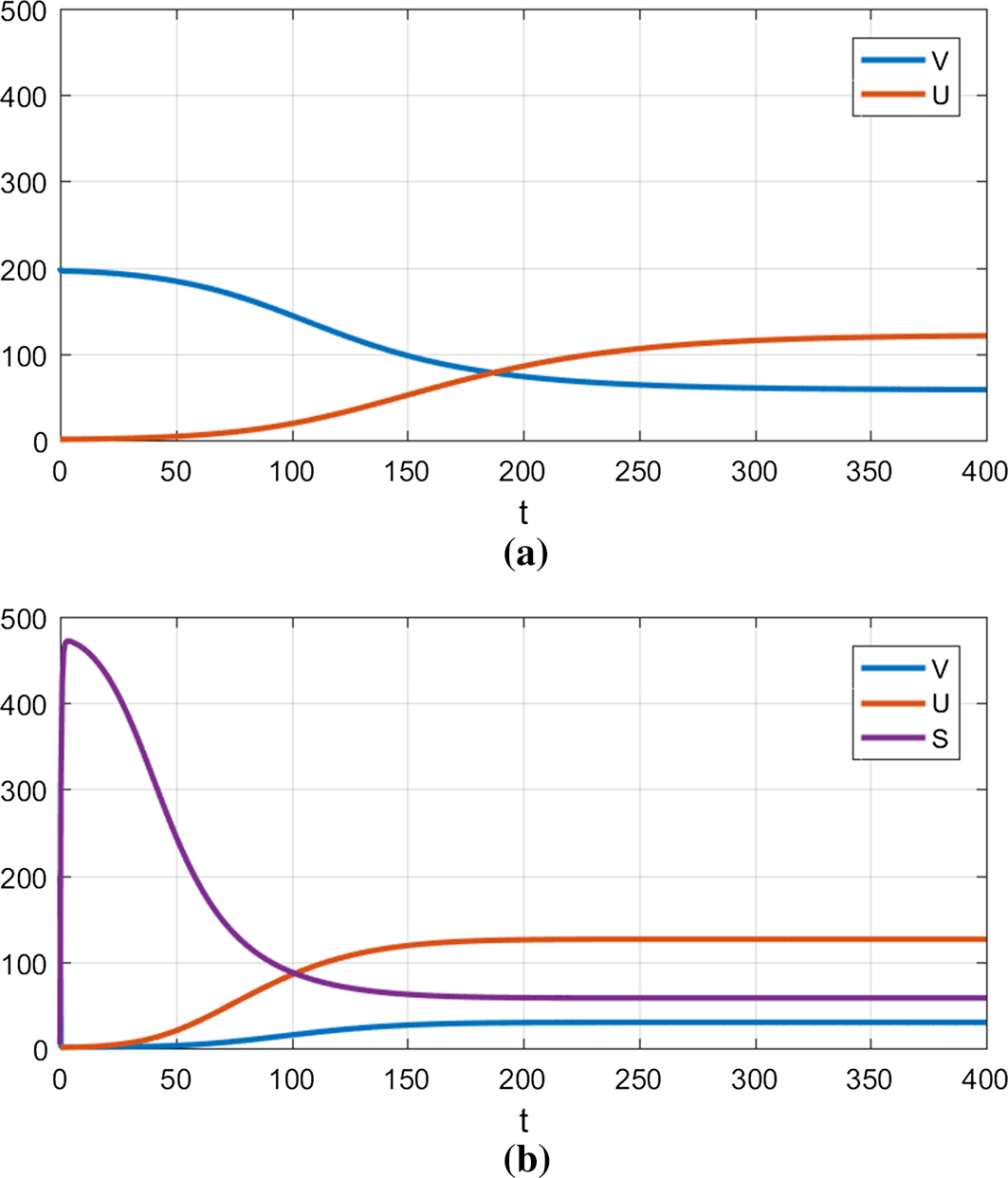
Numerical simulation using the nucleated polymerization model (**a**) and the template assistance model (**b**). The parameters used are described in Table 1 (Strain 1). The initial condition is given by $V(0)=\frac{\lambda }{\gamma }=200,U(0)=0.1, P(0)=(2x_0+\frac{\mu }{\beta })U(0), S(0)=0.2$. **a** Nucleated polymerization model, **b** Template assistance model

Because of its broad acceptance, it is crucial that the qualitative behavior of the nucleated polymerization model be maintained after modification. A generic study case is presented in Fig. 6, comparing the dynamics of the classical model with our model. One can see the overall dynamics of aggregate formation are qualitatively and quantitatively very similar for long times. Even though it is not shown, the evolution of *P*(*t*) also coincides with the one observed in the nucleated polymerization model for long times. The behavior during early times is dramatically different, because a new species is introduced. Our focus here is not on transient dynamics because we lack the data to study them. This could be a potential way to discriminate between different models. This shows that our model brings new possibilities without eliminating previously supported behavior (Greer et al. 2006; Prüss et al. 2006; Masel et al. 1999), especially on aggregate formation, size and numbers.

#### The template assistance model exhibits co-stability

**Fig. 7:**
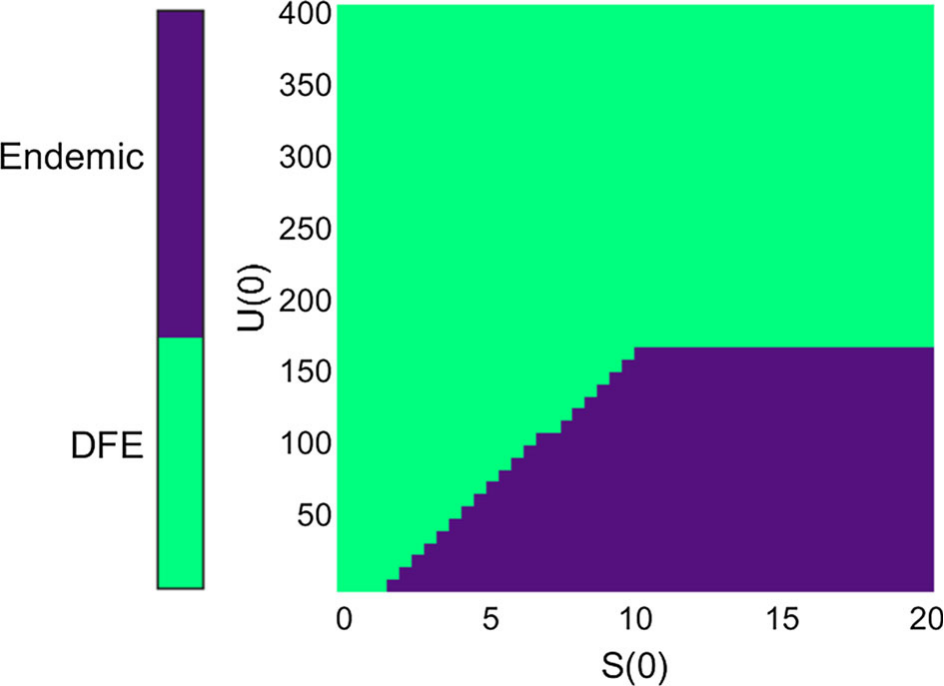
Representation of the basins of attraction of the endemic steady-state (dark color) and the disease-free steady-state (lighter color), according to the initial condition. The horizontal axis represents the initial number of subunits, and the vertical axis represents the initial number of aggregates. The parameters used are those of Strain 1 in Table 1, the initial value for *V* is set to $\lambda /\gamma$ (disease-free value), and the initial value for *P* is set to $(2x_0 +\mu /\beta )U(0)$ (steady-state mean size)

Even though the nucleated polymerization dynamics can be reproduced, our model offers a new variety of behaviors even for the single strain case. Because the disease-free equilibrium remains locally stable, our system exhibits dependency on the initial conditions. Figure 7 represents the basins of attraction of the endemic steady-state and the disease-free steady-state, for the case studied in Fig. 6. This helps to visualize the complexity and the diversity of behaviors allowed by our model. A biological interpretation for the shape of the basins plotted in Fig. 7 would be that an outbreak of prion propagation is possible only by having a specific initial mix of subunits and aggregates. In particular, starting off with too many aggregates (*U*(0) high) would prevent the subunits pool from successfully building up. We want to stress out that the important aspect of Fig. 7 is the shape of the basins, the precise values for the borders are irrelevant due to the arbitrary choice in parameters. Furthermore, this shape is subject to change when modifying the parameters, although so far we have no way of predicting these changes analytically. A numerical investigation could be undertaken, but in order to make biologically relevant predictions we require experimental observations or experimentally derived biochemical parameter values (see Sect. 4.2).

#### The two-strain case exhibits coexistence and co-stability

**Fig. 8:**
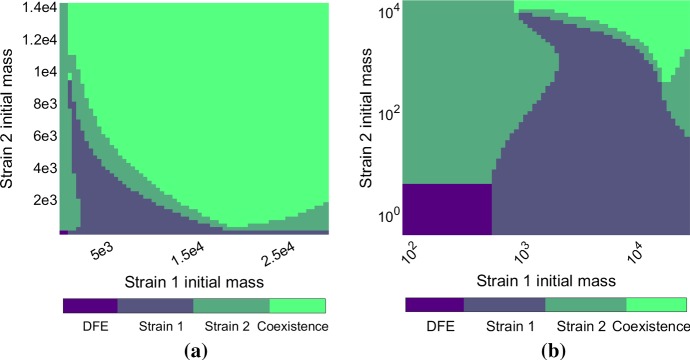
Representation of the basins of attraction of the different equilibria in the two-strain case, in linear scale (**a**) and logarithmic scale (**b**). The parameters used are described in Table 1. Each strain is initialized with its steady state proportions (see Sect. 2.1) and diluted with a specific rate (between 1 and $1.10^{-5}$), and *V* is initialized to the disease-free value $\lambda /\gamma$. The axes represent the initial mass of each strain, *i.e.* the initial value of $P+S$ for each strain (Strain 1 on the horizontal axis, Strain 2 on the vertical axis). The colors represent the outcome of the simulation (with colorbar above), one strain only or the two strains stably coexisting. Notice that the disease-free equilibrium is observed only in the bottom left corner (a) Linear scale (b) Logarithmic scale

To support the analytical results on strain coexistence (Sect. 3.4) and give numerical evidence of co-stability we show some simulations with two strains. Figure 8 illustrates the different possible outcomes, depending on the initial conditions. The set of parameters used (see Table 1) allows for four different equilibria to be co-stable (disease-free equilibrium, two single strain equilibria and coexistence equilibrium). Depending on the initial mass of each strain, the outcome can be the takeover of one strain or the stable coexistence of both, or the extinction of both. The shapes of the different basins of attraction are complex and non-intuitive. Indeed, in some cases increasing the initial amount of one prion strain can lead to its extinction (see the right-hand side of Fig. 8b). Although such behavior seems highly complex, we note that coexistence and co-stability are phenomena supported by experimental studies on mammalian prions (Langenfeld et al. 2016).

## Discussion

In this work, we have developed a novel model of prion aggregate dynamics which, in contrast to the traditional model in the field, is capable of supporting recent biological observations in both yeast and mammalian prion systems (Le Dur et al. 2017; Strbuncelj 2009). While a lack of detailed experimental findings have made precise quantitative comparisons impossible, the qualitative analysis of our model provides insight into a number of prion phenomena as we discuss below.

### Qualitative insight into open problems in prion biology

Our model suggests a mechanism for prion template-mediated conformational change. The essential idea is to involve a small oligomeric subspecies as the main conformers, as supported by recent experimental results (Igel-Egalon et al. 2017, 2018). This change in the nucleated polymerization model induces qualitative behavior that could account for a number of so far unexplained features of prion propagation and transmission. For most of these aspects, the idea is that separation between the templating efficiency and the polymerization efficiency, associated with a non-linearity in the templating activity, can cause intricate outcomes. Some strains will be associated with high numbers of subunits but low numbers of aggregates, or *vice-versa*. Most importantly, strain behavior would be modified depending on the other strains present and the initial configuration of aggregates versus subunits. Overall, judging from analytical and numerical investigation, we hypothesize that our model provides new and useful insight into prion dynamics. In particular, while our model was designed specifically to investigate mammal prion phenomena, similarities with yeast prions are indisputable. The general idea of a more complex polymerization pathway could apply profitably to the case of yeast prions. In particular, different oligomer pathways have been uncovered for yeast prions (Sharma et al. 2017), which suggests that our model could be modified to study yeast prion strains. The aspects of prion dynamics that could be investigated with our model include, but are not limited to, the following elements.

**Fig. 9:**
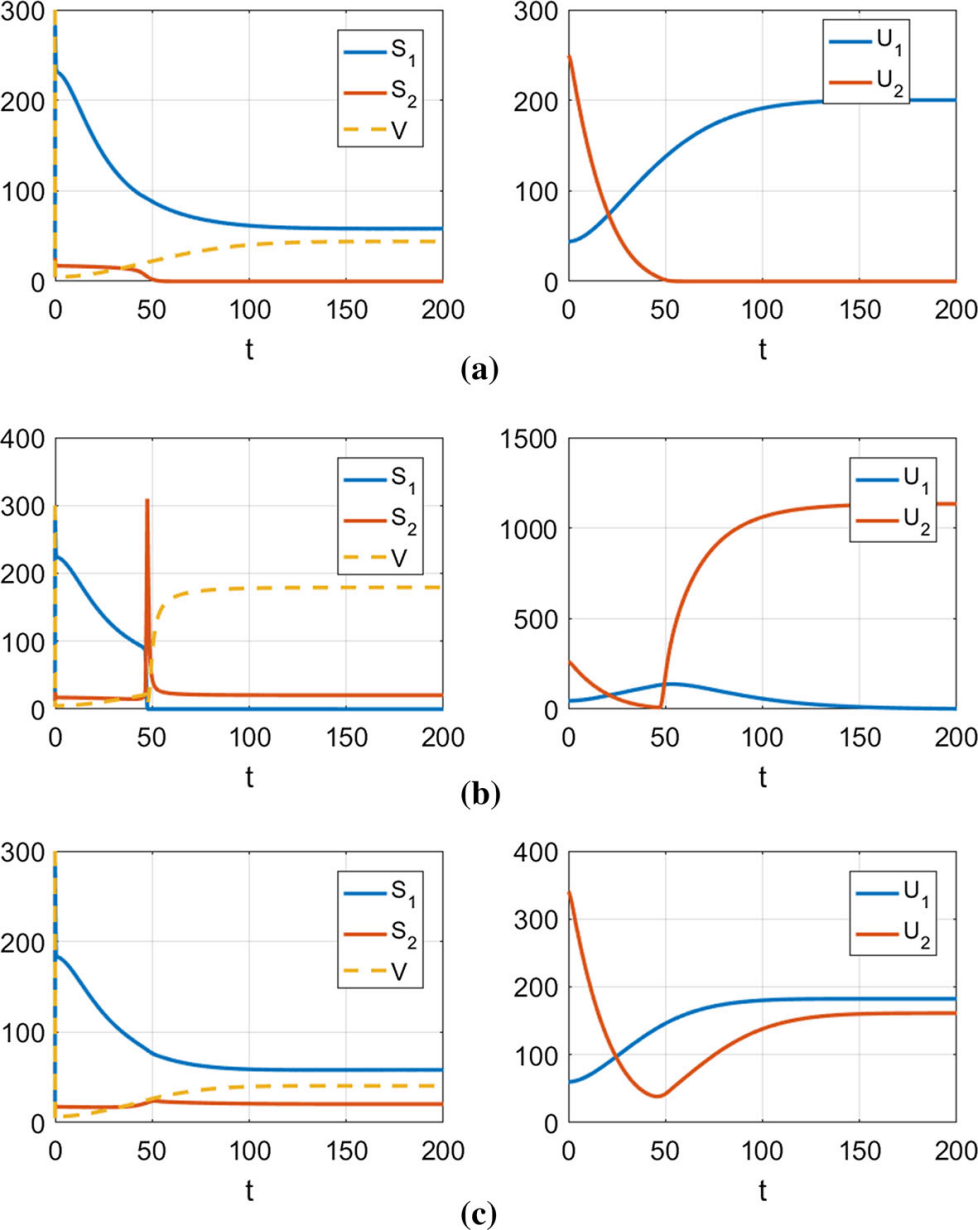
Illustrations of typical cases from the diagram shown in Fig. 8, representing the time evolution of the two strains (blue line is Strain 1, orange line is Strain 2). The parameters used are shown in Table 1. In each case, *V* is initialized to the disease-free steady-state $\lambda /\gamma$, and the strains are initialized with the specified conditions. The top panel (**a**) shows takeover of Strain 1, the middle panel (**b**) shows takeover of Strain 2, whereas the bottom panel (**c**) shows asymptotic coexistence of both strains **a**$(S_1(0),U_1(0),P_1(0))=(12.8,44.0,6398)$ and $(S_2(0),U_2(0),P_2(0))=(4.5,249.5,3095)$, **b**$(S_1(0),U_1(0),P_1(0))=(13.4,46.0,6689)$ and $(S_2(0),U_2(0),P_2(0))=(4.7,260.9,3235)$**c**$(S_1(0),U_1(0),P_1(0))=(17.5,60.0,8725)$ and $(S_2(0),U_2(0),P_2(0))=(6.1,340.3,4220)$

*Strain coexistence* is made possible by the consideration of a new prion species, as we emphasize in Figs. 8 and 9 and in Sect. 3.5. Indeed, under favorable environment configuration and initial conditions, two strains can coexist stably. This a major improvement compared to the nucleated polymerization model, which does not allow for coexisting strains in a robust parameter range, the competition for normal monomers being too strict (see Sect. 2.2). As was observed for the Creutzfeldt–Jacob Disease, and other prion associated disorders, multiple strains or substrains (Li et al. 2010) may be present simultaneously in a host (Langenfeld et al. 2016). It remains to be investigated quantitatively if the coexistence exhibited by our model can indeed correlate with the levels of prion coexistence *in vivo*. Coexistence of strains was also observed in yeast prions (Strbuncelj 2009), and our model shows that including an additional step in the polymerization pathway would give a reasonable explanation for this behavior. Other ideas have been suggested for the case of yeast prion strains, in particular the influence of molecular chaperones (Davis and Sindi 2016b).

*Inoculum influence* is another new feature of this model that could explain various experimental results of sub-propagation. The basins of attraction shown in Figs. 7 and 8 can only give a glimpse of how complex our model’s behavior really is. The intricateness of the different basins suggests that a slight variation of inoculum (i.e., introduced aggregate composition) and initial state could yield dramatically different outcomes, even more so when multiple strains are present. Changing slightly the initial proportions between the two strains can yield any of the three possible outcomes (one or the other strain taking over, or both strains coexisting). These outcomes in our model are supported by different experimental studies (Langenfeld et al. 2016; Le Dur et al. 2017) as we develop in Sect. 2.2. Overall it seems biologically relevant that the inoculum heavily influences the outcome, which is not the case with the classical nucleated polymerization model (see Sect. 2.3).

*Monomer expression level* is a factor that influences strain selection and sub-propagation (Le Dur et al. 2017). Our model does exhibit qualitatively different behaviors for different levels of monomer synthesis, as suggested by the bifurcation analysis in the single strain case, see Fig. 4. We also observed qualitatively different behavior in the two strain behavior when modifying the monomer source rate $\lambda$. In addition, for yeast a stable prion strain could be made unstable by increasing the rate of monomer synthesis. Although this has been attributed to balance between monomers and chaperones (Derdowski et al. 2010; Davis and Sindi 2016b), our template assistance model provides another possible hypothesis. We plan to investigate the dependency on the monomer source rate in future studies.

*Strain interaction* could be investigated with our model. Prior experimental studies reported that coinfection or superinfection by different prion strains can yield various and remarkable results. For instance Shikiya et al. (2010) and Langenfeld et al. (2016) sequentially inoculate different strains in a single host. Depending on the delay between two inoculations and the concentration ratio between the two inocula, the second strain can be totally suppressed or both strains can be made to coexist. Based on numerical studies, we suggest that such behavior could be explained with our model. For instance, disturbing a single strain equilibrium by adding a small inoculum of another strain can yield various results, blocking the new strain or eliminating the existing strain. This behavior is illustrated in Fig. 8 (in particular the bottom-right corner). Similar behavior has been observed in yeast prions (Bradley et al. 2002), and this suggests once again that the scope of our model could extend to the case of yeast prions.

*Decorrelation between aggregation and infectivity rise* is a feature reported numerous times in experimental studies (Rubenstein et al. 1991; Mays et al. 2015). It has been observed that the infectivity level of a prion-infected host’s brain can become significantly high before any detection of aggregated mass. The behavior of the template assistance model could account for such a decorrelation. Indeed with this model, the formation of aggregates is always preceded by a transitional phase with a very high number of subunits and almost no aggregates, as shown in Fig. 6. This transitional phase would not be detected by classical PrP^Sc^ monitoring methods, because of the low stability of these subunits. During this phase, the infectivity could already be high because of the high number of subunits. This transient phase could also account for the non-null delay observed even in seeding experiments, as suggested before (Alvarez-Martinez et al. 2011; Hingant et al. 2014). This raises some critical questions for future study as we discuss below. Finally, in yeast prions, the notion of propagon could be related to this idea (Derdowski et al. 2010). The minimal infectious agent, the propagon unit, would be a proper mix of aggregates and subunits.

### Challenges and limitations

In order to compare the numerical results we observe with *in vivo* behavior, we must be able to infer realistic values for the model parameters. Few studies have investigated parameter inference before (Masel et al. 1999; Derdowski et al. 2010), and then only in the case of a single strain. As such, more detailed experimental studies involving single and multiple strains will provide valuable data for refining parameter values.

However, before we can hope to fit parameters, it must be clear how to link biological observables (i.e., prion phenotypes) with quantities in our mathematical model. What biologists observe and quantify are global properties related to the disease (incubation time, deposition pattern, molecular density distribution, migration patterns, see Morales 2017 for a detailed description), but it is not clear how these properties are linked to the bio-chemical kinetics of aggregate formation. Accumulating studies suggest that phenotypes (disease properties) are not necessarily linked to prion strains (conformational states of the protein) in a one-to-one relation. Some phenotypes could potentially be composed of a mixture of different strains, and in some cases, strains are propagated “silently” (not influencing the phenotype) (Le Dur et al. 2017). This makes it even more complicated to determine each strain’s characteristics.

Finally, by introducing a novel species (prion subunits) we have continued to complicate the question of what is the *infectious agent* of prion phenotypes. It is not clear what causes disease and what causes the onset of symptoms. Some insight from our primary study would be that infectivity might be optimal when the inoculum consists of a specific mix of aggregates and subunits. Disturb these proportions and the propagation fails. Our work also suggests that these proportions are strain-specific, and dependent on the environment or on the presence of other strains.

### Ecological parallel and other modeling ideas

The coexistence of strains allowed by our model is similar to the phenomenon of “predation-mediated coexistence” in ecology. First, in the basic model (Sect. 2) the aggregates, *predators*, compete for the same resource, *monomers*. As such, they cannot coexist which is coherent with the competitive exclusion principle in ecology. In our model (Sect. 3), the aggregates are still predators but they do not compete for the same subunits, the equivalent of *preys*. These subunits do compete for a shared resource, but the predation limits their proliferation and thus their resource uptake. This allows different strains of subunits to coexist (as long as they are in presence of aggregates). This type of coexistence was exhibited in some ecological systems, for instance the *Daphnia* planktons (Declerck and Meester 2003; Gliwicz and Wrzosek 2008).

Judging from this rationale, other ideas could potentially yield the same outcomes, namely coexistence and co-stability. One idea would be to bound the monomer uptake of aggregates by including a non-linear aggregation rate in the equations of the nucleated polymerization model (see Sect. 2.1). Indeed if the polymerization speed saturated with high numbers of aggregates, we could potentially obtain coexistence. It could for example model the fact that the aggregates are in a constrained volume and their probability to encounter free monomers goes to 0 as their numbers grow. In the case of mammal brains or yeast cells, the aggregate densities at play are very low and the available volume is not limiting. This hypothesis is not relevant in our biological context, and this is why we do not develop it here.

An other idea is related to the biology of yeast prions. In yeast, the propagation of prions, specifically the fragmentation process, has been shown to be dependent on different molecular chaperones, including Hsp104 (Jones and Tuite 2005; Kryndushki et al. 2003; Winkler et al. 2012). While chaperones have not been identified as part of *in vivo* mammalian prion propagation, it is possible that they may be involved (Romanova and Chernoff 2009). Davis and Sindi (2016b) modeled a chaperone-limited fragmentation process, and numerically observed the possibility of coexistence of different strains. We intend to explore such possibilities in future studies, in particular for the case of yeast prions.

## Conclusion

We first presented the natural generalization of a classical model of prion dynamics in the context of multiple strains. We have shown that it does not reasonably explain the different experimental behaviors and phenomena associated with prion strains. We have then suggested a more complex mechanism of aggregate formation. Our model involves a novel misfolded species termed subunits, and allows for a new variety of behaviors. In particular, we show analytically that coexistence of different strains and co-stability of different equilibria are possible under our model. This suggests interpretations for experimental results where prion strains can interact, coexist or out-compete each other, mainly using the dependency on initial conditions and the presence of bifurcations.

In this work, most of our results are qualitative, but we plan to dedicate future work to quantitative investigation of this model in comparison with experimental results. To do so, it may be necessary to consider different formulations of the model, including discrete aggregate sizes but also stochastic simulations. More experimental results will be needed in order to conduct such a study. In addition, during our mathematical investigation, other ideas came up that could be potential ways of modeling multiple strain interaction. We chose the simplest idea that was consistent in the biological context of focus, namely mammalian prions *in vivo*. We were driven towards simplicity by the lack of data, but we are confident that collaboration with biologists will allow for quantitative studies and adjustments to the model.

## A Proof of Theorem 1

For the existence and uniqueness of solutions we refer to Prüss et al. (2006), from which the results can easily be extended to the general *N* strain case.

### A.1 Global stability of the DFE

When ${\mathscr {R}}_0=\max \nolimits _{i=1\ldots N}{R_0^i}\le 1$, we define the function *L* given by

$$\begin{aligned}&L(V,U_1,P_1\ldots , U_N,P_N)=\frac{1}{2}\Big (V-\frac{\lambda }{\gamma }\Big )^2\\&\quad +\,\sum _{i=1}^N{b_i\Big (\frac{\mu _i}{\beta _i}U_i+P_i\Big )},\ \text {with }b_i=2\frac{(\mu _i+\beta _i x_i)^2}{\beta _i \tau _i}-\frac{\lambda }{\gamma }-\frac{\beta _i^2 x_i^2}{\beta _i \tau _i}. \end{aligned}$$

This function is a Lyapunov function for the DFE $(\lambda /\gamma ,0,0,\ldots ,0,0)$. Indeed it is positive, because $R_0^i\le 1$ implies $\frac{(\mu _i+\beta _i x_i)^2}{\beta _i \tau _i}\ge \frac{\lambda }{\gamma }$ and thus $b_i\ge \frac{(\mu _i+\beta _i x_i)^2-\beta _i^2 x_i^2}{\beta _i\tau _i} >0$ for $i=1\ldots N$. It evaluates to 0 at the DFE, and its derivative along trajectories is

$$\begin{aligned} \dot{L}&=\Big (V-\frac{\lambda }{\gamma }\Big )\Big (\lambda -\gamma V -\sum _{i=1}^N(\tau _i VU_i+\beta _i x_i^2U_i)\Big )-\sum _{i=1}^N b_i U_i\Big (\frac{\mu _i^2}{\beta _i}+2\mu _i x_i-\tau _i V+\beta _i x_i^2 \Big ),\\&= -\,\frac{1}{\gamma }\Big (V-\frac{\lambda }{\gamma }\Big )^2-\sum _{i=1}^N\tau _i U_i \Bigg (V^2-V\Big (\frac{\lambda }{\gamma }+\frac{\beta _i x_i^2}{\tau _i}+b_i\Big )+x_i^2\frac{\lambda \beta _i}{\gamma \tau _i}+b_i\frac{(\mu _i+\beta _i x_i)^2}{\tau _i\beta _i} \Bigg ). \end{aligned}$$

The choice of $b_i$ yields

$$\begin{aligned} \dot{L}&= -\,\frac{1}{\gamma }\Big (V-\frac{\lambda }{\gamma }\Big )^2-\sum _{i=1}^N\tau _i U_i \Bigg (V^2-2V\frac{(\mu _i+\beta _i x_i)^2}{\beta _i \tau _i}+\Big (\frac{(\mu _i+\beta _i x_i)^2}{\tau _i\beta _i}\Big )^2\\&\quad +\,\frac{\mu _i(\mu _i+2\beta _i x_i)}{\beta _i\tau _i}\Big (\frac{(\mu _i+\beta _i x_i)^2}{\beta _i\tau _i}-\frac{\lambda }{\gamma }\Big )\Bigg ),\\ \dot{L}&= -\,\frac{1}{\gamma }\Big (V-\frac{\lambda }{\gamma }\Big )^2 -\sum _{i=1}^N\tau _i U_i \Bigg (\Big (V-\frac{(\mu _i+\beta _i x_i)^2}{\beta _i \tau _i}\Big )^2\\&\quad +\,\frac{\mu _i(\mu _i+2\beta _i x_i)}{\beta _i\tau _i}\Big (\frac{(\mu _i+\beta _i x_i)^2}{\beta _i\tau _i}-\frac{\lambda }{\gamma }\Big )\Bigg ). \end{aligned}$$

It is simple to verify that, under the hypothesis that $R_0^i\le 1$ for each strain, the derivative of *L* is negative along the trajectories, and evaluates to 0 at the DFE only.

### A.2 Global stability of the endemic steady-state

As in the Theorem 1, let us assume all strains have distinct $R_0^i$ values, and that Strain 1 has the maximal value ${\mathscr {R}}_0=R_0^1=\max _{i=1\ldots N} R_0^i$. It is convenient for this part to consider a modification of the model, namely choosing to study $W_i=P_i-x_i U_i$ instead of $P_i$ for each strain. The equations of the model now read

$$\begin{aligned} \frac{dV}{dt}&=\lambda -\gamma V(t) +\sum _{i=1}^N{(-\tau _i V(t)U_i(t)+\beta _i x_i^2 U_i(t))}, \\ \frac{dU_i}{dt}&= \beta _i W_i(t) -(\mu _i+\beta _i x_i) U_i(t),\ i=1\ldots N,\\ \frac{dW_i}{dt}&=\tau _i V(t)U_i(t)-(\mu _i+\beta _i x_i) W_i(t),\ i=1\ldots N. \end{aligned}$$

In this system, the disease steady-state (involving Strain 1) is given by $V^*=\frac{(\mu _1+\beta _1 x_1)^2}{\beta _1\tau _1}$, $U_1^*=\frac{\lambda \beta _1\tau _1 -\gamma (\mu _1+\beta _1 x_1)^2}{\mu _1\tau _1(\mu _1+\beta _1 x_1)}$,$W_1^*=\frac{\mu _1+\beta _1 x_1}{\beta _1}U_1^*,\forall j\in \{2\dots N\}, U_j^*=P_j^*=0.$

When ${\mathscr {R}}_0=R_0^1=\max \nolimits _{i=1\dots N} {R_0^i}>1$, we define the function *L* given by

$$\begin{aligned}&L(V,U_1,W_1,\ldots ,U_N,W_N)\\&\quad =\Big (1+\frac{\beta _1 x_1^2}{\tau _1 V^*-\beta _1 x_1^2}\Big )(V-V^*-V^*\log (V/V^*))\\&\qquad +\frac{\mu _1+\beta _1 x_1}{\beta _1}(U_1-U_1^*-U_1^*\log (U_1/U_1^*))\\&\qquad +(W_1-W_1^*-W_1^*\log (W_1/W_1^*))\\&\qquad +\Big (1+\frac{\beta _1 x_1^2}{\tau _1 V^*-\beta _1 x_1^2}\Big )\sum _{j=2}^N(1-\frac{1}{\eta _j})(\frac{\mu _j+\beta _j x_j}{\beta _j}U_j+W_j),\\&\qquad \text {with }\eta _j=\frac{\frac{\beta _j^2 x_j^2}{\beta _j \tau _j}V^*+\Big (\frac{(\mu _j+\beta _j x_j)^2}{\beta _j\tau _j}-V^*\Big )\Big (\frac{(\mu _j+\beta _j x_j)^2}{\beta _j\tau _j}-\frac{\beta _j^2 x_j^2}{\beta _j \tau _j}\Big )}{\Big (\frac{(\mu _j+\beta _j x_j)^2}{\beta _j x_j}-\frac{\beta _j^2 x_j^2}{\beta _j \tau _j}-V^*\Big )^2}\text { for }j=2\dots N. \end{aligned}$$

This function is a Lyapunov function for the disease-steady state mentioned above. First it is positive, recalling that the classical function

$$\begin{aligned} \phi : \xi \rightarrow \xi -\xi ^*-\xi ^* \log (\xi /\xi ^*) \end{aligned}$$

for $\xi ^*>0$ is positive on $\mathbb {R_+^*}$, with a global minimum of 0 in $\xi =\xi ^*$. Notice also that $\tau V^*>\beta _1 x_1^2$. The terms in the sum are also all positive, because for $j\in \{ 2\dots N\}$, $\eta _j>1$. Indeed, the numerator of $\eta _j$ can be expressed as

$$\begin{aligned}&\underbrace{\Big (\frac{(\mu _j+\beta _j x_j)^2}{\beta _j x_j}-\frac{\beta _j^2 x_j^2}{\beta _j \tau _j}-V^*\Big )^2}_{\text {denominator of }} \eta _j + V^*\Big (\frac{(\mu _j+\beta _j x_j)^2}{\beta _j \tau _j}-V^*\Big )\\&\quad +\, \frac{\beta _j^2 x_j^2}{\beta _j \tau _j} \Big (\frac{(\mu _j+\beta _j x_j)^2}{\beta _j \tau _j}- \frac{\beta _j^2 x_j^2}{\beta _j \tau _j} \Big ). \end{aligned}$$

The fact that Strain 1 has the highest $R_0^i$ value means it is associated with the lowest $\frac{(\mu _i+\beta _i x_i)^2}{\beta _i \tau _i}$. With the formulation above, this proves that for all $j=2\dots N$, $\eta _j >1$ and thus $1-\frac{1}{\eta _j}>0$. It is easy to verify that *L* evaluates to 0 at the point of the disease steady-state.

To compute the derivative of *L* along the trajectories, recall that $\frac{d}{dt}\phi (\xi )=\frac{\xi -\xi ^*}{\xi }\frac{d\xi }{dt}$. Straightforward calculations lead to, after simplifications,

$$\begin{aligned}&\dot{L}(V,U_1,W_1,\ldots ,U_N,W_N)=\\&\quad -\,\Big (\gamma +(\gamma +\tau _1 U_1)\frac{\beta _1 x_1^2}{\tau _1 V^*-\beta _1 x_1^2}\Big )\frac{(V-V^*)^2}{V}\\&\quad +\,\tau _1 V^*U_1^*\left( 3-\frac{V^*}{V}-\frac{W_1}{W_1^*}\frac{U_1^*}{U_1}-\frac{V}{V^*}\frac{U_1}{U_1^*}\frac{W_1^*}{W_1}\right) \\&\quad -\,\Big (1+\frac{\beta _1 x_1^2}{\tau _1 V^*-\beta _1 x_1^2}\Big )\sum _{j=2}^N\tau _j \frac{U_j}{V}\frac{1}{\eta _j}\Bigg (V^2+V\bigg (\eta _j\Big (\frac{(\mu _j+\beta _jx_j)^2}{\beta _j \tau _j}-V^*-\frac{\beta _j^2x_j^2}{\beta _j \tau _j}\Big ) \\&\quad -\,\frac{(\mu _j+\beta _jx_j)^2}{\beta _j \tau _j}\bigg ) +\eta _j\frac{\beta _j^2x_j^2}{\beta _j \tau _j}V^*\Bigg ). \end{aligned}$$

Details of the calculations are not shown but can be made available. The first term in this expression is obviously negative along trajectories. The second term can be studied through the function defined on ${\mathbb {R}}_+^*\times {\mathbb {R}}_+^*$

$$\begin{aligned} (x,y)\rightarrow 3-x-y-\frac{1}{xy}. \end{aligned}$$

This function is negative if $x+y>3$ or if $\frac{1}{xy}>3$. The complementary of this region is closed and bounded, and the function being continuous on this region, it admits a maximum value. Canceling the gradient leads to the only extremum $x=y=1$ where the function evaluates to 0. If we now replace *x* by $\frac{V}{V^*}$ and *y* by $\frac{W_1}{W_1^*}\frac{U_1^*}{U_1}$, we have shown that the second term in $\dot{L}$ is negative along trajectories, with a maximum value of 0 being reached at $V=V^*,U_1=U_1^*$ and $W_1=W_1^*$. The last term remains to be studied, especially the second order polynomials in *V*. For $j\in \{2 \dots N\}$, the polynomial in the *j*-th term of the sum can be expressed (using the choice for $\eta _j$):

$$\begin{aligned}&V^2+2\frac{\frac{\beta _j^2 x_j^2}{\beta _j \tau _j}V^*}{ \frac{(\mu _j+\beta _j x_j)^2}{\beta _j \tau _j} - \frac{\beta _j^2 x_j^2}{\beta _j \tau _j} -V^*}V\\&\quad +\, \frac{\beta _j^2 x_j^2}{\beta _j \tau _j}V^* \frac{\frac{\beta _j^2 x_j^2}{\beta _j \tau _j}V^*+\Big (\frac{(\mu _j+\beta _j x_j)^2}{\beta _j\tau _j}-V^*\Big )\Big (\frac{(\mu _j+\beta _j x_j)^2}{\beta _j\tau _j}-\frac{\beta _j^2 x_j^2}{\beta _j \tau _j}\Big )}{\Big (\frac{(\mu _j+\beta _j x_j)^2}{\beta _j x_j}-\frac{\beta _j^2 x_j^2}{\beta _j \tau _j}-V^*\Big )^2}V. \end{aligned}$$

Its discriminant is given by

$$\begin{aligned} \varDelta&=4\frac{\frac{\beta _j^2 x_j^2}{\beta _j \tau _j}V^*}{\Big (\frac{(\mu _j+\beta _j x_j)^2}{\beta _j \tau _j} - \frac{\beta _j^2 x_j^2}{\beta _j \tau _j} -V^*\Big )^2}\\&\quad \times \, \Bigg ( \frac{\beta _j^2 x_j^2}{\beta _j \tau _j}V^* - \bigg (\frac{\beta _j^2 x_j^2}{\beta _j \tau _j}V^*+\Big (\frac{(\mu _j+\beta _j x_j)^2}{\beta _j\tau _j}-V^*\Big )\Big (\frac{(\mu _j+\beta _j x_j)^2}{\beta _j\tau _j}-\frac{\beta _j^2 x_j^2}{\beta _j \tau _j}\Big ) \bigg ) \Bigg ), \\&=-\,4\frac{\frac{\beta _j^2 x_j^2}{\beta _j \tau _j}V^*}{\Big (\frac{(\mu _j+\beta _j x_j)^2}{\beta _j \tau _j} - \frac{\beta _j^2 x_j^2}{\beta _j \tau _j} -V^*\Big )^2}\Bigg (\frac{(\mu _j+\beta _j x_j)^2}{\beta _j\tau _j}-V^*\Bigg )\Bigg (\frac{(\mu _j+\beta _j x_j)^2}{\beta _j\tau _j}-\frac{\beta _j^2 x_j^2}{\beta _j \tau _j}\Bigg ). \end{aligned}$$

For the same reasons as before, this quantity is negative. The polynomial thus has no real roots, and is always positive. This proves that each term in the sum in $\dot{L}$ is positive. Overall, we have shown that every term in derivative is negative along trajectories and they cancel out at the point of the disease steady-state. This concludes the proof of the global stability of this equilibrium.
